# The three main monotheistic religions and gm food technology: an overview of perspectives

**DOI:** 10.1186/1472-698X-9-18

**Published:** 2009-08-22

**Authors:** Emmanuel B Omobowale, Peter A Singer, Abdallah S Daar

**Affiliations:** 1 Department of English, University of Ibadan, Ibadan, Nigeria; 2 McLaughlin-Rotman Centre for Global Health, University Health Network, Toronto, Canada; 3 Dept of Medicine, University of Toronto, Toronto, Ontario, Canada; 4 Dalla Lana School of Public Health, Unversity of Toronto, Toronto, Canada

## Abstract

**Background:**

Public acceptance of genetically modified crops is partly rooted in religious views. However, the views of different religions and their potential influence on consumers' decisions have not been systematically examined and summarized in a brief overview. We review the positions of the Judaism, Islam and Christianity – the three major monotheistic religions to which more than 55% of humanity adheres to – on the controversies aroused by GM technology.

**Discussion:**

The article establishes that there is no overarching consensus within the three religions. Overall, however, it appears that mainstream theology in all three religions increasingly tends towards acceptance of GM technology per se, on performing GM research, and on consumption of GM foods. These more liberal approaches, however, are predicated on there being rigorous scientific, ethical and regulatory scrutiny of research and development of such products, and that these products are properly labeled.

**Summary:**

We conclude that there are several other interests competing with the influence exerted on consumers by religion. These include the media, environmental activists, scientists and the food industry, all of which function as sources of information and shapers of perception for consumers.

## Background

In 1999, the Church of England issued a statement that "religious traditions, which are reservoirs of wisdom accumulated and sifted over the centuries, have a vital role to play in helping society to reach the right conclusions" about the genetic modification (GM) of food crops [1]. We know that public acceptance of GM food technology is a crucial issue in the field. Whilst public acceptance is rooted, in part, in religious views, to our knowledge the views of different religions and their potential influence on consumers' decisions have not been systematically examined in a single overview article. In view of the interest and controversy generated by GM food technology, we present a brief overview of relevant positions articulated by religious leaders representing different faith communities and secular commentators – academics, especially scholars who also double as experts on issues relating to these three religious traditions, scientists and adherents of these monotheistic religions – on GM food technology. Specifically, we focus on the world's three major monotheistic religions: Judaism, Islam and Christianity, whose adherents, who mostly live in developing countries, collectively constitute more than 55% of the world population.

## Discussion

In recent years, the genetic modification of food crops has become a controversial issue in global trade and development [2]. A Genetically Modified Organism (GMO) is one whose genetic structure has undergone a deliberate re-engineering or alteration. For the purpose of this paper, the process involves the introduction of a foreign gene that enables the host organism to manifest specific qualities conferred by the gene [3-5].

Since the introduction of the genetically engineered Calgene's Flavr Savr tomato into the American market in the early 1990s [6], a wide range of new GM food crop products have been developed and marketed worldwide [7]. Not surprisingly, the reception of these new food products has been mixed. Some of the criticisms of GM food technology focus on risks to the environment [8], risks to human beings who consume them [9] the possibility of a few multinational companies dominating global food production [10], and the marginalization of farmers in developing and developed countries [11]. Other complaints include the possibility of dependence of Southern countries on the industrialized North for food [12], the loss of the genetic originality of plants and crops from different parts of the world as a result of gene engineering [13], the distortion and destruction of the cell structure of these organisms [14] and improper labeling [15], especially when GM crops and non GM crops are mixed together [16].

Despite these criticisms, in many parts of the world, numerous scholars, governments and international agencies have been consistent in voicing their support for GM food technology. They argue that scientists alter the genetic structures of plants in order to confer beneficial properties on them. Such benefits include the enhancement of the quality and quantity of crops to increase their micronutrient content [17], the reduction in the maturation time of seedlings [18], the enhancement of plant resistance to pests and disease [19], the improvement of the adaptability of crops to nutrient deficient soil [20] and the production of proteins for human and animal medicine [21] and the conferment of drought resistance.

The idea that we humans should not be "playing God" is widely held among people of many backgrounds. In the context of GM crops, the idea that transgenesis and the crossing of species barriers constitute "playing God" is obviously a subject worthy of serious attention, even if it cannot be upheld with serious analysis. We perhaps need to refer to ethicists for a more objective analysis of this subject. Bernard Rollin, an ethicist, posits that there is nothing intrinsically wrong with scientists crossing the species barrier given that so many of the world's "moral categories" have been adapted or displaced to meet the challenges of our technologically driven contemporary world [22]. Although, many ethicists would not agree with this argument, Rollin's position is consistent with the official position of the UK government, articulated in the Polkinghorne Report, which states that whatever gene (whether human, animal or plant) that is integrated into a host genome is in fact a laboratory fabricated version of the original and its development is not a contravention of ethical, cultural, religious norms or social codes [23]. Polkinghorne was both a scientist and a clergyman and we believe that his views, and the views of the committee he chaired, represent a fair analysis of both the science and ethical and moral dimensions of the subject when the report was written in 1993.

The early controversy seems to have settled where policy is concerned, and a number of safeguards have been proposed to mitigate the risks mentioned above. The European Union (EU), while accepting GM food technology, requires that every food product produced from a genetically modified organism must be labeled accordingly [24]. The position of the World Health Organization (WHO), an organ of the United Nations, is that humankind "could benefit enormously from biotechnology, for example, from an increase in the nutrient content of foods, decreased allergenicity and more efficient food production" [25]. The WHO also argues that any technology involved in food production must be thoroughly evaluated to ensure that concerns about issues such as food, human health and the environment are addressed in a holistic and all-inclusive manner. The same point is emphasized in the Report of the African Union's high-level panel on modern biotechnology, "Freedom to Innovate" [26].

In addition to the positions of international agencies, governmental and non-governmental bodies on GM food technology, religious leaders have attempted to play a role in guiding consumers about GM food technology. For some people religion and the guidance of religious authorities continues to exert a powerful influence on cultural and ethical conventions, especially in developing countries, where research viewed by scientists as being purely scientific and experimental, may be considered inimical and threatening to people's religious convictions and practices.

### Perspectives on GM technology in Judaism, Islam and Christianity

#### Judaism

Within Judaism, the interpretation of life is based on the postulations of different Rabbis, whose moral authority stems from their in-depth understanding of the Divine as contained in the *Torah*, the Hebrew bible, in response to questions of social significance [27]. In a 2005 commentary on GM food technology, Esra Galun, a respected Jewish Professor of Plant Sciences at the Weizmann Institute of Plant Sciences, who is an expert on Jewish religious prescriptions on plants and food crops recognizes that determining whether it is good to develop genetically modified food crops is fraught with problems [28]. Galun refers to two other Jewish philosophers and religious scholars, E. Goldschmidt and A. Maoz, who submit that, based on Jewish religious laws and traditions, the development of transgenic plants by researchers are permissible if they are not directly prohibited by God and if the research will benefit mankind. Another Jewish Rabbi, Akira Wolff [29], supports this view when he states that Jewish tradition believes that man was created in God's image and this affords him the opportunity of partnering with God in the perfection of everything in the world. According to him, Jewish law (*Halacha*) accepts genetic engineering to save and prolong human life as well as increase the quality or quantity of the world's food supply. On the biblical prohibition of *Kilayim*, or mixing of different species of animals and plants, Wolff believes that God does not prohibit the genetic modification of food crops. In concluding his paper, Wolff states "man may manipulate the creation (of God) ... [but] all the legally permitted actions must bring the world closer to perfection and not further away".

In contrast, Michael Green, a British based Jewish commentator, who espouses Orthodox Judaism, argues that there is no consensus within Judaism about GM food technology and he cites a prominent Jewish environmental group in the United States, the Teva Learning Centre (TLC), to support his position. The TLC believes that the GM food technology is a violation of *Kilayim*, the mixed breeding of crops or livestock [30]. Green also refers to two bible verses, Leviticus 19:19 and Deuteronomy 22:9–11, where God prohibits the mixing of species, as proofs that God made "distinctions in the natural world", which Jews must not breech by eating GM food or engaging in GM food research. Green believes that genetic engineering in its entirety endangers nature and human beings. Similarly, in a paper published in 2000, a Conservative Jewish Rabbi, Lawrence Troster, argues that religious traditions should be more cautious before endorsing genetically modified foods. He calls for an acknowledgement of humankind's "limitations in the face of the depth and grandeur of the order of creation" [31].

The different positions on the issue of GM food technology and GM food products and how they affect the average Jew is also discussed in an article entitled "Are Genetically Modified Foods Kosher?" [32], written by Rabbi Tzvi Freeman. Freeman explicitly states that the controversy about whether Jews can eat GM food or engage in GM research stems from the postulations of two renowned Jewish Rabbis, Moshe Ben Nachman and Yehuda Lowe. According to Freeman, Nachman, a medieval Rabbi, argues that God has given humankind the right to dominate and use any of God's creation "but not to disturb its fundamental nature". However, Lowe, who wrote his own interpretations of the *Torah *about three hundred years after Nachman, argues that "any change that human beings introduce into the world already existed in potential when the world was created. All the humans do is bring that potential into activity". Thus, while acknowledging the divergent Jewish positions on the modification of food crops, Freeman emphasizes the need for Jews to look at the health and environmental implications of GM food technology and through such scrutiny seek answers to the question of whether their introduction into the human food supply is actually beneficial or detrimental to the environment and humankind.

The divergence in the views of these Jewish religious leaders, scholars and commentators shows that there is no universal agreement within Judaism on whether Jews can eat GM food products or engage in research in the area of GM food technology.

#### Islam

Islam is made up of two major branches, Sunni and Shia, distinguished by some doctrinal and historical differences [33]. However, despite these differences, the rulings on modern biological and technological issues tend to be quite similar [34]. At a seminar in Kuwait on genetics and genetic engineering in October 1998, a group of Muslim intellectuals concluded that although there are fears about the possibility of the harmful effects of GM food technology and GM food products on human beings and the environment, there are no laws within Islam which stop the genetic modification of food crops and animals [35]. The Islamic Organization for Medical Sciences in collaboration with the Islamic Fiqh Academy, Jeddah, the World Health Organization's Eastern Mediterranean Regional Office, Alexandria, and the Islamic Education, Science and Cultural Organisation (ISESCO) organized the seminar. Worthy of note is the involvement of the Islamic Fiqh Academy, which is an Academy for advanced study of Islam and which was established by the Organization of Islamic Conference (OIC) in 1988 and which is administered by a body of Islamic clerics. The above conclusion reflects the widely held views of most scientifically informed Muslim scholars, whether Sunni or Shia. Thus it is noteworthy that scientists in Islamic countries like Egypt and Indonesia (the world's largest Muslim country), are actively manipulating plant genes in a variety of ways. In fact, in 2003, the Indonesian Ulemas Council (MUI) approved the importation and consumption of genetically modified food products by Indonesian Muslims [36].

Ibrahim Syed, an Islamic cleric and the President of the Islamic Research Foundation International, an amalgamation of different Islamic religious groups, is regarded as a leading expert on the interpretation of the Quran in the light of recent advances in the area of modern technology [37]. He has written about the consensus among Muslim scholars that the *Quranic *verse forbidding man from defacing God's creation "cannot be invoked as a total and radical ban on genetic engineering ... If carried too far, it would conflict with many forms of curative surgery that also entail some change in God's creation" [38]. Syed enjoins African and Asian countries, with large Muslim populations, to "reject the propaganda of extremist groups" campaigning against genetic engineering and these new technologies and to embrace them wholeheartedly.

In her own contribution to the discourse, a female Muslim scholar, Fatima Agha al-Hayani, who has written and commented on several aspects of the Islamic religion, contends that Muslims must ensure that genetic modification "may remain mercy-driven" and promote righteousness [39]. She believes GM food technology has the ability "to carry God's work, alleviate hunger and suffering, secure justice and equity for everyone". Therefore, Muslims "must keep up with the new research and discoveries and make connections within the scientific fields". However, the different perspectives on GM food technology within the Muslim world are obvious in a letter written in October 2006 to the British government by Majid Katme, on behalf of the United Kingdom Islamic Medical Association. Katme, a highly respected personality within the Muslim community in the United Kingdom [40] quotes copiously from the *Quran *and asserts that there is no need for genetic modification of food crops because God created everything perfectly and man does not have any right to manipulate anything that God has created using His divine wisdom. He also states that the *Quran *contains several verses, prohibiting man from tampering with God's creation. He ends the letter by emphasizing the position of members of the United Kingdom Islamic Medical Association that there are no benefits that would accrue to Britain from GM food production [41]. Thus, even within Islam, there is no consensus by religious scholars and commentators on whether the *Quran *accepts genetic modification of food crops and the consumption of GM food products by Muslims.

#### Christianity

The Catholic Church is the largest Christian denomination in the world [42], with all significant matters of theology and Canon Law decided within the Vatican, under the ultimate direction of the Pope [43]. Nevertheless, there is flexibility among various bishops and experts that are well tolerated within the greater Church so long as they do not conflict with fundamental teachings. Thus theological matters of social significance, such as GM crops, may follow different paths such as:

(1) a no "official" Vatican position;

(2) a limited "policy statement or interpretation of scripture or traditions;

(3)or formal theological positions, published in the form of Papal encyclicals developed by the Congregation for the Doctrine of the Faith, a Vatican-based body whose role is to provide formal interpretations in the case of socially relevant issues, such as abortion or euthanasia.

In 2003, the head of the Pontifical Council for Justice and Peace, based at the Vatican, Cardinal Renato Martino, asserted that the Catholic Church supports genetic modification of food crops as an answer for world starvation and malnutrition and because "scientific progress was part of the divine plan" [44,45]. Martino's statement aligns with a papal address by John Paul II in November 2000, in which he states the Vatican's support for the use of biotechnology in agricultural production as long as the "research is submitted beforehand to rigorous scientific and ethical examination". While Benedict XVI, who succeeded John Paul II as Pope, has condemned human genetic engineering, he has not made any categorical statements on GM food technology.

In 2001, the Pontifical Academy of Sciences, (PAS) an influential Catholic organization, published the proceedings of 2 conferences that it organized in 1999 and 2000 on the "Sciences and the future of Mankind". The PAS argues that it is imperative that new or modern technologies be developed to assist in the improvement of agriculture in developing countries as well as help in feeding the world's hungry people who are increasing daily as a result of the rapid expansion of the world's population. The organization is of the opinion that the genetic modification of crops is not a new phenomenon having been in existence for about 10,000 years. However, the organization also advocates for the close cooperation of scientists, governments and farmers to ensure that genetically modified crops are safe for human consumption, especially since modern science has developed novel means for detecting and removing allergens in crops. From the perspective of the PAS, the benefits of genetically modified crops are immense as they facilitate the actualization of the global goal and desire "to develop plants that can produce larger yields of healthier food under sustainable conditions with an acceptable level of risk" [46]. Recently, scientists at a 2009 conference organized by the PAS came to the conclusion that genetically modified crops "offer food safety and security, better health and environmental sustainability" as a solution to the hunger and poverty ravaging different parts of the world [47].

However, there are certain organizations within the church that are anti GM crops and who espouse positions that are different from the views of Cardinal Renato Martino and Pope John Paul II. These groups believe that the pro-GM lobby has been able to infiltrate the Vatican to enlist its support for the genetic modification of plants. One of such "dissident" groups is the St Columban's Mission Society, which is an Order of Catholic Priests. In recent times, the Columban society has criticized the Pontifical Academy of Science for cooperating with the US embassy to the Vatican to host a pro-GM conference entitled "Feeding the World: The Moral Imperative of Biotechnology". Father Sean McDonagh, an Irish Columban Priest and ecologist has been vociferous in arguing against the support of the Vatican and its Pontifical Academy of the Sciences for GM food technology. According to McDonagh, "All the experts at Catholic development agencies have taken the position that this is not the way to address food security, and that there's no magic bullet for hunger. What's needed is land reform, financial aid to small-scale farmers, markets where they can get value so they're not caught by the middle man. I've spent 40 years at this sort of work, and I know that's the way forward" [48].

The Church of England, which is also known as the Anglican Church, also avers that "human discovery and invention can be thought of as resulting from the exercise of God-given powers of mind and reason". In effect, scientists who are human beings are exercising their qualities as "images of God", who have been divinely endowed to intervene in "natural processes" [49]. The Church of England believes that genetically modified crops must be properly labeled so as to afford "consumers a legitimate degree of informed choice".

It is pertinent to note that there are also differences within the Anglican Church on the issue of GM food technology. While the worldwide head of the church, the Archbishop of Canterbury, is based in England, where he serves as the head of the church in England, there are branches of the Anglican Church in different parts of the world, mostly in countries formerly colonized by Britain. These national branches are very independent and the congregational meetings of the Presiding Archbishops of the different national branches in England, called the Lambeth Council, simply serve as a means of sustaining the links between these different branches of the worldwide Anglican Communion. In fact, the Archbishop of Canterbury is not in a position to impose the views of the English branch of the church on the other members of the Anglican Communion. A good case in point is a statement credited to a former Anglican Archbishop of Cape Town, Njongonkulu Ndungane, who argues against the introduction of GM foods not only in South Africa but throughout Africa. Ndungane is of the view that Africans do not need genetically engineered food. He believes that it is not safe for human consumption and the African farming systems. He asserts that GM food crops would lead to a reduction in jobs, increase African dependence on the countries of the North and destroy biodiversity [50].

In January 2002, the Conference of European Churches (CEC) presented the outcome of the critical examination of the genetically modified food controversy by its Church and Society Commission. The CEC comprises 126 churches, which belong to different Christian traditions (Protestant, Orthodox, Anglican and Old Catholic). The report shows that these Christian churches agree to the introduction of GM food technology on the premise that it is important to establish a "theology of creation" that properly balances research in the area of biotechnology with a genuine concern for everything created by God, which encompasses the whole of humanity and nature in its entirety [51]. The major highlight of the CEC report is its affirmation that the genetic alteration of plants is consistent with biblical teaching. The report further states that although nature belongs to God, it is not sacred and it can be manipulated for the benefit of humankind. What this suggests is that in the opinion of the theologians and scholars who wrote the report, GM food technology is acceptable, as long as scientists remain within specified ethical and moral limits.

Dialectically opposed to the position of the Conference of European Churches is another Christian ecumenical body, the World Council of Churches (WCC), which is based in Geneva. It is a fellowship of churches from more than 120 countries. In June 2005, its Working Group on Genetic Engineering of the Justice, Peace and Creation team published a document entitled "Caring for Life: Genetics, Agriculture and Human Life". The report concluded that it is unethical, from a Christian perspective, for scientists to dabble in the genetic modification of food crops. The position of the working group members is reflected towards the end of the document, where they aver that "GE messes with life, messes with truth, messes with our common inheritance (i.e. human culture and biodiversity), messes with justice, messes with human health, messes with the lives of peasant farmers in developing countries and the relationship between human beings and other forms of life" [52].

In the concluding segment of the article, Christians scientists who work for companies involved in genetic engineering and who believe in the bible's core message of truth and justice are enjoined to "become whistle-blowers and conscientious objectors" to any research in the field.

Our brief overview of religious perspectives about GM foods suggests that there is no overarching consensus on the permissibility of GM technology, performing of GM research, or consumption of GM foods within the world's three main monotheistic religious traditions. Overall however, it appears that mainstream theology in the world's monotheistic religions accepts the genetic modification of food crops, performing GM research and consuming GM foods as long as there is adequate scientific, ethical and regulatory scrutiny of research and development of such products, and they are properly labeled. The potential implications of such support for the genetic engineering of plants are diverse and range from increasing awareness in humankind's creative ingenuity as well as influencing government policies on issues like food security, international trade and the reduction of poverty.

In today's complex world, in spite of the pervasive presence of religious institutions, the ethos of life is gradually tilting towards individualism and materialism. Djamchid Assadi, a Professor of Marketing and Communication at the American University of Paris, argues that in this age where the manipulation of every aspect of nature by scientists is seen as a triumph and a celebration of humankind's intellectual achievements, religion is less influential in contemporary secular societies than it once was. According to Assadi, unlike the pre-modern period when religion constituted the prevailing ethos around which life revolved, the postmodern era is dominated by "rationalization, meaning the adoption of norms and values emphasizing effectiveness, efficiency and cost benefit equations ..." [53].

Thus, questions about the appropriateness of GM food technology that might once have been legislated upon by religious institutions may ultimately be settled by individual consumers, particularly those who face hunger and uncertain food security, while struggling to survive in a harsh, hostile, volatile and increasingly secular world, where life changing decisions are increasingly no longer being left alone in the esoteric world of the divine and the supernatural [54,55]. This is borne out by the work of Ferdaus Hossain and Benjamin Onyango [56], who contend that the information provided by governments, the media, industry and scientists on biotechnology confuses the consumers. In a survey they conducted to determine consumer acceptance of nutritionally enhanced genetically modified foods, they discover that it is how the individual consumer perceives the risks and benefits of GM crops based on sundry sources of information that actually determines the acceptance or non-acceptance of GM food products. Other studies on consumer acceptance of GM crops [57-60] also echo this view.

In a recent publication, Arthur Einsele [61] has observed a gap between science and perception with regards to GM food products. He concludes that most people have very little understanding of the general facts of what genetic engineering entails and argues that the benefits of GM food technology should be made "literally visible". He posits that people would have to realize the benefits of the genetic modification of food crops before they can accept it. Consumers must also be made to understand, in a "factual, user friendly" manner, that some of the adverse consequences of GM food technology, suggested by its opponents, have not materialized.

## Summary

Based on our analysis, we reach these conclusions: First, there is no consensus on whether GM food technology should be banned or accepted by the religious groups discussed. Second, there is also no monolithic view of beliefs within each religion with respect to GM food technology, a situation, which gives room for different interpretations of issues. Third, there is no agreement on what should be prescribed for the followers of each religion with regards to GM food products and the comments by the religious leaders are intended to simply provide guidance about GM food technology. Fourth, competing with the influence exerted on consumers by religion are several other interests like the media, environmental activists, scientists and the food industry, all of which function as sources of information for consumers. Thus, these religions, while assisting adherents in forming opinions, can only be one of the many factors that can be expected to influence consumers' decisions on GM food technology.

## Competing interests

The authors declare that they have no competing interests.

## Authors' contributions

EBO, PAS and ASD jointly participated in conceiving the study and developing its content. All authors read and approved the final manuscript.

## Authors' informations

EBO is a Senior Lecturer in the Department of English, University of Ibadan, where he teaches Bioethics and different aspects of literature. He is currently spending his sabbatical at the McLaughlin-Rotman Centre for Global Health, University of Toronto.

ASD is a Professor of Public Health Sciences and of Surgery at the University of Toronto, and is Senior Scientist and Director of the Program on Ethics and Commercialization at the McLaughlin-Rotman Centre for Global Health, University Health Network and University of Toronto, Toronto, Ontario, Canada.

PAS is Professor of Medicine at the University of Toronto, and is Senior Scientist and Director of the McLaughlin-Rotman Centre for Global Health, University Health Network and University of Toronto Toronto, Ontario, Canada.

## Pre-publication history

The pre-publication history for this paper can be accessed here:

http://www.biomedcentral.com/1472-698X/9/18/prepub
